# A qualitative study of stakeholder views of the conditions for and outcomes of successful clinical networks

**DOI:** 10.1186/1472-6963-12-49

**Published:** 2012-02-28

**Authors:** Elizabeth McInnes, Sandy Middleton, Glenn Gardner, Mary Haines, Maggie Haertsch, Christine L Paul, Peter Castaldi

**Affiliations:** 1 Nursing Research Institute, St Vincents and Mater Health Sydney and Australian Catholic University, National Centre for Clinical Outcomes Research, DeLacy Building, St Vincent's Public Hospital Darlinghurst, 379, Victoria Road, Darlinghurst 2010, NSW, Australia; 2 Royal Brisbane & Women's Hospital, Bowen Bridge Rd, Herston 4029, QLD, Australia; 3 Queensland University of Technology, 2 George, St Brisbane 4000, QLD, Australia; 4 Sax Institute, Level 2, 10 Quay Street, Haymarket NSW, Australia 2000; 5 University of Sydney, Camperdown 2006, NSW, Australia; 6 University of Newcastle, School of Medicine and Public Health University Drive, Newcastle 2308, NSW, Australia

## Abstract

**Background:**

Clinical networks have been established to improve health outcomes and processes of care by implementing a range of innovations and undertaking projects based on perceived local need. Limited research exists on the necessary conditions required to bring about successful network outcomes and what characterises network success from the perspective of those involved in network initiatives. This qualitative study identified stakeholder views on i) the conditions for effective clinical networks; and ii) desirable outcomes of successful clinical networks.

**Methods:**

Twenty-seven participants were interviewed using face-to-face audio-recorded semi-structured interviews. Transcribed data were coded and analysed to generate themes relating to the study aims.

**Results:**

Five key factors represented as sub-themes were identified as important conditions for the establishment of successful clinical networks under the main theme of *effective network structure, organisation and governance*. These were: building relationships; effective leadership; strategic evidence-based workplans; adequate resources; and ability to implement and evaluate network initiatives. Two major themes encapsulated views on desirable outcomes of successful clinical networks: *connecting and engaging *which represented the outcomes of interdisciplinary and consumer collaboration and, partnerships with state health and local health services, and *changing the landscape of care*, which represented the importance of outcomes associated with improving services, care and patient health outcomes and implementing evidence-based practice.

**Conclusions:**

This study provides new knowledge on the conditions needed to establish successful clinical networks and on desirable outcomes arising from network projects and initiatives that are considered to be valuable by those working in or associated with clinical networks. This provides health services with information on what needs to be in place for successful networks and on the types of outcomes that can be considered for assessing network effectiveness.

## Background

Internationally, clinical networks have emerged over the last ten years as an important clinician-driven innovation for attaining system-wide improvements in healthcare delivery and patient outcomes [1]. Commonly, the remit of clinical networks is to identify how and where improvements in health service delivery and patient outcomes can be made in the particular specialty represented by the network and to implement changes in association with key government health organisations. Important features of clinical networks are that they can provide a structure for liaising across institutions, allowing greater clinical input into models of service delivery [2,3]; provide 'bottom up' views on the best ways of tackling complex healthcare problems[4] and are usually multidisciplinary involving doctors, nurses, allied health professionals, scientists, managers, and consumers [1]. Clinical networks can also provide an organisational vehicle for embedding evidence-based care into health systems and engaging clinicians to change practice in line with evidence [3,5,6].

The term clinical network has been used to describe many variants of networks ranging from fully integrated service delivery systems to informal communities of practice [7]. In this paper the term refers to networks of voluntary clinicians[8] that aim to improve clinical care and service delivery using a collegial approach to agree and implement a range of strategies. Unlike managed networks, they do not have a formal service delivery function involving the organisation and co-ordination of all clinical services that the patient needs through primary, secondary and tertiary care [9]. In Australia, in 2004, the Agency for Clinical Innovation (then the greater Metropolitan Clinical Taskforce, GMCT) led the development and establishment of voluntary collegial clinical networks in New South Wales. The purpose of these networks is to provide opportunities for clinicians from a range of healthcare professions, and consumers to participate in the design and implementation of models of care and clinical plans; to develop and implement a range of projects to foster evidence-based practice and to provide clinicians with a structure that facilitates research and service delivery across local health care boundaries. These networks generally meet after hours and strongly rely on voluntary involvement of clinicians and consumers and have support from a Network Manager to assist with the co-ordination of network projects and network administration. The ACI clinical networks are commonly chaired by clinicians or consumers and are multidisciplinary in focus, involving doctors, nurses, allied health professionals, managers, and consumers. The remit of ACI networks is to identify how and where improvements can be made in the particular specialty represented by the network and to implement changes in association with key government health organisations in order to improve health service delivery and patient outcomes. Drawing on these experiences, clinical networks with similar purposes are currently being implemented in other Australian states. Examples of network initiatives include provision of education for implementation and workforce initiatives; development of policies, clinical guidelines and consumer resources; implementing processes for monitoring safety and quality.

Studies from a number of countries have shown that while there is some empirical evidence of positive impact on processes of care,[10-12] there is uncertainty around what constitutes 'successful' network outcomes and what the factors, or conditions, that are required to help networks achieve their outcomes [13]. While there are studies that have investigated the factors that affect the establishment of voluntary clinical networks,[7,8,14] there are no studies that have researched what those connected to networks think is required to establish a successful network. Because clinical networks operate within a complex political, cultural and organisational context, research to identify conditions for successful networks from the perspective of those involved in networks can provide useful data for organisations wishing to establish future networks and maximise success in achieving outcomes.

In addition, there is limited empirical evidence demonstrating the benefits of networks to healthcare consumers and health services [13]. This field of research could benefit from stronger evaluative designs and knowledge of which outcomes common to a range of networks (ie other than disease-specific patient health outcomes) should be measured. Identifying outcomes that are regarded as important by those connected to networks and policy-makers, and that could inform the measurement of outcomes across networks with different clinical foci would benefit this field of research. Taking account of stakeholder perspectives helps to ensure that a future set of outcome measures has utility and content validity [15]. There are no studies that have investigated the views of those who work in or who are connected with networks about desirable outcomes. Clearly articulating the desired outcomes of networks can provide insights into ways network effectiveness can be measured.

The aims of this study were to identify key stakeholders' views on the conditions required to establish successful and effective clinical networks and also their views on desirable outcomes of successful networks.

## Methods

### Research Design

To answer the research questions of a) what do stakeholders identify as necessary conditions for establishing successful clinical networks and b) what do stakeholders identify as outcomes of successful clinical networks, a qualitative research design was chosen in order to obtain a full range of authentic participant views and for distilling key themes in this under-researched area [16,17].

### Participants

A purposive maximum variation sampling approach was used to recruit participants from each of four groups, that is who were either directly involved as a participating clinician or involved directly or indirectly at a policy or strategic level. More detail about the definition of the four stakeholder groups: network drivers, network participants, Senior health service managers and Senior policy-makers, is provided in the list below:

**Network drivers**: individuals with detailed knowledge about the activities of at least one established clinical network, specifically network managers who had been in position at a network for a minimum of two concurrent years and current and past Network Co-chairs and Governing Committee members.

**Network participants**: participants in network activities and meetings as defined by records of attendance (medical staff, allied health professionals, nurses, and consumers) involved in networks that had been established for a minimum of two years.

**Senior health service managers**: senior managers at executive management level who held a clinical operations or clinical governance role at a hospital or from one of the eight (as there were at the time the study was conducted) metropolitan Area Health Services (AHS) that had clinicians involved in networks. Individuals in this group included senior local health service executives such as the general manager, clinical director, chief executive and director of clinical operations.

**Senior policy-makers**: executive level policy makers that worked in organisations with a relationship to the Clinical Network Executive (that is, network administrative body). Examples of these organisations are those with a statewide strategic role in: health policy and planning; quality improvement, patient safety initiatives; developing clinical guidelines and protocols and professional development.

Those in the network driver and network participant groups had to have been associated with a network that had been established for a minimum of 2 years in order to capture informed perspectives from those with at least some experience of working in or establishing networks. In addition to the criteria outlined above, eligible individuals were those aged 18 years of age or over and able to give informed consent.

We recruited participants from each of the four groups by asking the Clinical Network Executive Office (the main administrative office of the then GMCT), to identify those who met the eligibility criteria. Once selected, all potential participants were sent an advanced letter that explained the research aims and informed them that they met the eligibility criteria and that a researcher may later contact them to see if they are agreeable to participate. A phone call was then made one week after to four individuals in each participant group who were selected from the list (not in alphabetical order) provided by the Clinical Network Executive Office and appointments made for the interview. The initial number of individuals was selected to ensure that a range of views was captured. Additional participants were selected one at a time to ensure maximum variation. All individuals contacted consented to participate. The final sample size of 27 was reached by saturation of themes, that is, no new insights were identified in the data. Nine participants were from the network driver group; six were network participants; four were senior health service managers and eight were senior policy-makers. Those from the network driver and network participants groups (called 'network groups' below) were associated with 10 of the 20 networks established at the time the research was conducted.

### Data collection

Individual semi-structured face-to-face interviews using a topic guide were conducted at the interviewee's place of work. The interviews were of 30-60 minutes duration and were audio-recorded. Participants were asked for their views on what they thought were the most important things that needed to be in place (that is, conditions) for clinical networks to be successful and to achieve positive outcomes. To elicit views on 'conditions' participants were prompted to nominate facilitators, or 'things that need to be in place' for successful networks to be established. Participants were also asked for their views on what they thought were desirable outcomes resulting from network initiatives or projects. In this context, the term 'clinical networks' referred to networks of clinicians that aim to improve clinical care and service delivery using a collegial approach to agree on and implement a range of strategies. Participants were asked to give their general views rather than to limit their responses to their direct experiences with any particular clinical network(s).

### Data analysis

Interviews were anonymised and transcribed verbatim to produce transcripts of narrative text for thematic analysis. The factors of interest, namely conditions and outcomes, provided a framework for the initial categorisation of text. Thematic analysis with open coding was performed whereby each segment of interview text that related to the factors of interest was coded as a provisional theme. These codes were descriptive and linked with representative examples from the original text and the same code was assigned to data that represented similar themes. The process of generating themes was inductive. Themes were identified, coded, recoded and classified by examining regularities, convergences and divergences in the data. The themes and sub-themes derived reflect the language used by the participants. Refinement of our analysis led to both themes and sub-themes, the latter being conceptually linked to the main themes. We also highlighted differences and convergences in responses from stakeholder groups. For 'conditions' the resulting codes were organised into five sub-themes and on the basis of these, an overarching theme was identified: Effective Network Structure, Organisation and Governance. For 'outcomes' the resulting codes were organised into four sub-themes and two overarching themes were identified: Connecting and Engaging and Changing the Landscape of Care. Participant narratives have been used to illustrate meaning in the themes and summaries.

In order to ensure the quality of results, two researchers conferred on the analysis separately coding text and generating themes and sub-themes and then comparing output. In addition, the results were circulated to participants to check that the findings had fidelity with their perceptions and experiences. Following this, minor adjustments were made. There was good concordance between researchers in the analysis of jointly reviewed transcripts and validation by participants did not show disagreement with the analysis.

### Ethics approval

Ethics approval was gained from the Human Research Ethics Committee of the Australian Catholic University. All participants were informed of the objective of the study and that they were free to participate or withdraw from the study at any point. Participants gave written consent to be interviewed and for the interviews to be audio-recorded. Recordings and transcripts were coded so that the origin of each one could not be identified.

## Results

### Conditions for effective networks

The main integrating theme that emerged was Effective Network Structure, Organisation and Governance. This theme reflects participant feedback that pointed towards successfully established networks having effective structure, organisation and governance. The sub-themes discussed below are conceptually related to this main theme in that they represent the conditions that help to realise Effective Network Structure, Organisation and Governance. These sub-themes, namely *building relationships*; *leadership*; *strategic evidence-based workplans*; *adequate resources*; and *ability to implement and evaluate network initiatives *were identified by participants as the most important conditions.

#### Building relationships

All participants spoke of the value of building relationships within and external to networks. Well-established and well-functioning networks were said to be characterised by a commitment to 'engagement, networking and partnerships' and this involved the building of a critical mass of clinicians, consumer and stakeholders:

"The thing about the networking is that it's actually driven by a combination of clinicians, senior influential clinicians, and the managers they work with. If you've got one without the other then you can't act effectively, you can't make things happen."

Less successful and effective networks were seen by all stakeholders as being less well organised and structured and as being run by 'turfdom and fiefdoms' focused on objectives of individual interest rather than of broader relevance to the health system:

"Trying to protect their particular practice and fear that they're going to lose something. Rather than seeing the gains for everybody else in the state."

Reaching non-networked clinicians and consumer groups, rather than only reaching 'the converted' through targeted communication strategies was considered important by all stakeholder groups in order to maximise the impact of networks:

"Even in the big teaching hospitals - there will be a lot of people who wouldn't know what [the network] was."

Rotating chairs between clinical disciplines was regarded as a strength by those in the network groups and as an effective strategy for building local support, because it 'gets away from the doctor-boss type thing' and signals that the network values the contribution of all disciplines. The need for appropriate representation in working groups and on governing committees was also seen as important for the same reason:

"If people saw it as being just the teaching hospitals driving this then our district hospital and our GP services wouldn't want to be a part of it. So you've got to have an appropriate level of representation."

In addition, developing relationships with the rural and remote health care sector was nominated by all stakeholder groups as a key condition, although networks were strongly identified with metropolitan areas:

"If we really want this to work, the network should be NSW [wide]; it should have been inclusive from the beginning."

In terms of external relationships, the engagement of the state health department, the minister of health and chief health officer was uniformly seen by all participants as a fundamental condition for establishing a successful network:

"Department of Health's engagement is pivotal and we have networks that have fantastic relationships with the chief health officer and with an engaged Minister". [the NSW]

Some from the senior health service manager and policy-maker groups, expressed that there were some tensions in the evolution of this relationship and this was said to be a barrier to network development. It was felt that not all network personnel effectively liaised with the state health department. This was largely attributed to a lack of understanding of the correct procedures and processes for consulting and communicating with government.

However, from the network group perspective, feedback was centred on the 'slowness of the state health bureaucracy' and that it was a 'challenge to feel that we are listened to and able to influence policy'. These factors were felt to hamper the establishment and progress of network projects and was an ongoing source of frustration:

"They'll participate but they'll do it at their speed when it's convenient for them and how they want to do it. I think that attitude is what prohibits things getting done."

Developing strong positive relationships between networks and senior administrators and executives from local health services were regarded as essential by all stakeholder groups. However, the development of this relationship was seen as a 'work in progress'. From the perspective of those in the network group, this was partially attributable to local health services reluctance for network initiatives to extend across health service boundaries:

"This is part of the local health service culture - they see all health needs being able to be met within their boundaries and by the health service employee."

From the perspective of some in the Senior health service manager and Senior policy-maker groups, there was a view that some networks currently exclude health service managers input into projects and governance leading to a perception that some networks had developed 'in silos':

"They are integrated vertically but not horizontally."

It was felt that local health service manager representation on network committees would ensure that networks deliver projects of relevance to the region covered by the health service and help to increase health service and local clinician buy-in to networks. One Senior health service manager went further stating that networks should be formally embedded in clinical streams at the local health area and hospital level, otherwise momentum would be lost after initial establishment of a network and clinicians would 'drift away' from the network. Formalisation in this way was seen as helping to strengthen the governance and organisation of networks.

#### Effective leadership

All stakeholder groups saw leadership across three different levels as an essential building block for effective structure, organisation and governance, and as a major facilitator of network effectiveness and success. Having 'a strong network manager who can direct a lot of the stuff' with ability to effectively liaise with clinicians, consumers, and external stakeholders; implement workplans; and effectively run network operations was one level of leadership which was seen as important, particularly for setting up organisational and governance processes. A participant from the network driver group summed up the importance of the network manager's role as follows:

"It's a big jigsaw puzzle and you have to have one person, I think, who knows all the pieces of the puzzle."

Influential and passionate clinical leaders (who lead or chair a network) were also regarded as necessary to build effective networks. Characteristics of a good clinical leader included being well respected by the clinical community and as having 'a bit of fire in their belly' and 'a fair bit of ambition'. The ability to keep people engaged and to influence a wide range of consumers and clinical stakeholders was identified by all groups as an essential part of developing an effective network:

"You need to keep people's passion; if you lose that you lose sight of everything."

A clinical leader who is both transformational (that is, able to deal with complex change and be an inspirational and visionary champion of the network) and transactional (that is, have planning and organisational skills) was seen as the ideal by those in the Senior policy maker and health service manager groups:

"If you don't have leaders that are modelling those behaviours, it doesn't percolate down through the rest of the network."

The third level of leadership thought necessary for establishing successful networks centred on the role of the Clinical Network Executive. This body was seen by all groups as having the authority and credibility to 'take management issues up the chain' to state government and as having a direct line to both the Minister of Health and the Director-General of NSW Health. From the perspective of those in the network driver group, maintenance of that direct line was seen as critical to the ongoing sustainability of networks. Some from this group commented that having a co-ordinating body such as the Clinical Network Executive helped to 'keep [networks] going and on track':

"The support from the Executive is very, very important and executive understanding of the issues is even more important. I don't think that we could undertake our projects without [the Executive Officer] who really is on top of all the issues and knows the ways to address them."

For some in the Senior Policy-Maker group it was also felt that the Clinical Network Executive role helped to build alliances between clinicians and managers. According to one participant, this level of leadership and overall network governance, helped to narrow what was seen as a 'yawning gulf between clinicians and managers'.

#### Strategic evidence-based workplans

A strategic, feasible evidence-based workplan with a vision and measurable milestones was seen by all stakeholder groups as an important facilitator of network success and as enabling networks to demonstrate a strategic role in the broader health context. The importance of workplans aligning with state health government priorities was particularly stressed by Senior health service manager and policy-maker groups who saw this as a key factor in attracting 'buy-in' and fostering positive relationships with government agencies. Some participants from these groups expressed that networks which have an 'alternative agenda' to the priorities of the health department resulted in 'poor' and ineffective network development because the priorities of networks were then isolated from 'the big picture'. Furthermore, the 'selective use of evidence' to underpin or justify network workplan objectives was seen by some from these groups as indicative of a network without a strategic focus and one that is more inclined to be dominated by self-interest:

"Because the clinicians will always tell you... we're evidence based and all this, but if it doesn't fit their model suddenly it's not necessarily good evidence."

However, according to participants from the network groups, as well as addressing state health priorities, network workplans also need to be 'valuable to the participants' and reflect their concerns and interests in order to keep clinicians engaged:

"It's not just a question of a goal that NSW Health thinks is achievable. It needs to be able to address the concerns of clinicians who are giving up their time."

Across all stakeholder groups there was a view that there was insufficient emphasis in network workplans across the full continuum of care, with primary care and the needs of the rural and remote health care sectors and populations 'rarely getting a look-in'. This was seen as compromising the impact of network initiatives and acknowledged to be related to lack of resources to fund such initiatives.

#### Adequate resources

For those in the network groups, access to adequate resources, staff, administrative assistance, technical support and information technology was seen as integral to the success of a network and being able to develop feasible workplans. In the view of some participants from these groups, inadequate resources meant that some networks were struggling to deliver outputs, thus diminishing the impact of clinical networks on health service improvement and patient outcomes:

"The really good things that could be done is fantastic and you can't do that if you don't properly resource."

A dedicated network manager was said to be essential and where more than one network was assigned to one network manager this was regarded as a significant barrier to successful operation and ability of the networks to deliver on their workplans:

"You can't obviously put 100 per cent into each of the three of them, so it just makes it that much more difficult to really achieve your outcomes"

Reliance on clinicians' voluntary time, was cited as a problem, leading to burnout, unless time was given within working hours to participate in network activities. Participants in all groups commented that time release by health services for clinicians and clinical leaders to participate in network activities was needed. The lack of funding for technical expertise, such as data managers and IT assistance, as well as for software and training, was also seen by several participants from all groups as hindering successful development and was seen as limiting the ability of the networks to evaluate and monitor changes as a result of network projects. However, a participant from the Senior policy-maker group felt that networks could make savings by targeting activities to priority health needs and ensuring that areas for which there is the most evidence for practice and service change are addressed.

#### Power to implement and evaluate

An important condition nominated by those from the network groups was for networks to be mandated to implement changes in practice or service delivery as a result of network innovations. That this had not yet occurred was a source of considerable frustration for many in the network groups:

"We come up with fantastic guidelines, policies, frameworks, clinical protocols, recommendations. But then who does the network take it to? Who does it go to? It can't implement it."

For example, some in the network groups stated that they had been involved in developing new models of care but implementation of these was a challenge:

"You might have a really good project, a really strong project, lots of clinical input, very strong recommendations, but those recommendations aren't taken up by the Department of Health."

Some in the network groups mentioned that not having 'power or teeth to implement changes' placed networks at risk of losing the enthusiasm of involved clinicians. The need for networks to be empowered to make changes was also acknowledged by some in the Senior policy-maker group: 'need to sort out who is responsible for implementation. Feedback from the Senior manager group highlighted that to facilitate implementation, networks needed to garner the support of local health services to implement and ensure that projects and innovations are clinically relevant.

In addition, for all groups, being able to evaluate the impact of networks on health service delivery and patient outcomes was seen as critical for securing further funding and establishing the credibility of networks as an effective health care organisation. Lack of resources and skills to evaluate impact of network projects was seen by those in the network groups as jeopardising long-term sustainability because of not being able to empirically demonstrate improvements arising from network projects.

### Outcomes of successful clinical networks

Thematic analysis revealed two main themes *connecting and engaging *and *changing the landscape of care *- each with sub-themes that represented a range of desirable network outcomes specific to those themes. The two sub-themes for *connecting and engaging *were 1) interdisciplinary and consumer collaboration and, 2) partnerships and engagement with state health and local health services. The two sub-themes related to *changing the landscape of care *were i) improving services, care and patient health outcomes and, 2) implementing evidence-based practice.

### Theme 1: Connecting and engaging

#### Interdisciplinary and consumer collaboration

Interdisciplinary and consumer collaboration was highly valued as a desirable outcome by those in network groups as it was seen as facilitating 'new ways of working', and providing 'a voice for all disciplines'. These new ways of working in turn, promoted the sharing of knowledge and development of collaborations for improving patient outcomes:

"Nurses and allied health particularly, have felt, probably to start with, intimidated by the process but now feel equal partners in it. So, that is a sign of success to me, before you even get to patient care."

The development of interdisciplinary and consumer collaboration were thought by many participants across all stakeholder groups to have shifted some clinical groups from a stance that was often 'competitive, defensive and protective' to a more 'co-operative and collegial ethos'. This ethos was said to have led to 'a lot of renewed optimism' in working in health care, which was also cited as a desirable outcome by one participant in the network participant group. Another network participant from a well-established network, regarded the role modelling by senior clinicians of these new ways of collegial working as important an outcome as achieving improvements in patient outcomes:

"Above all else, role modelling, so that younger clinicians can see that all disciplines are conversing, working together and can see that everyone has a place and a voice at the table. This is the most important, most desirable network outcome in my view."

Involving consumers and having consumer representation in networks were also regarded as a desirable outcome. As a participant from the network driver group said: 'A network is successful if it brings together health care professionals and consumers even before any patient outcomes are achieved'.

#### Partnerships with state health and local health services

Partnerships with external stakeholders, such as the state government health department and the local health services, were regarded as an important and desirable outcome by all stakeholder groups, not least because it was seen as giving networks credibility and legitimacy in the broader health arena. Participants stated that since the formation of clinical networks, overall the relationship between clinicians and government agencies had improved and had led to greater mutual understanding of perspectives:

"I think the successful outcomes have been the partnerships and how those partnerships have worked in an open and transparent way and that's how we need to do it. At a hospital level, at a local health service level, at a state level and at a national level."

A health minister and NSW Health Director-General who would 'listen and implement network initiatives' were considered as 'big outcomes' by those in the network groups, as was achieving allied health representation within the state health department:

"There was not an allied health representative at NSW Health until clinical networks started. So, that is a sign of success to me, before you even get to patient care."

Open and transparent partnerships with local health services was also considered an important outcome, though seen as a 'work in progress' by both those involved in networks and senior health service managers. For participants from the Senior health service manager and policy-maker groups, a valuable outcome would be the involvement of networks in local health service planning and the development of clinical plans.

### Theme 2: Changing the landscape of care

#### Improving services, care and patient outcomes

Across the spectrum of stakeholder groups, the overall impetus for participation in network activities and establishment of networks was to work for the patient cohort and to improve the effectiveness of clinical services. This could be done through network projects that add value to existing services by becoming involved in service planning and improving the delivery of services. This was expressed in comments such as: *'removing the waste in the system'*; and '*breaking down the barriers to the provision of care for patients in NSW'*.

**Figure 1:**
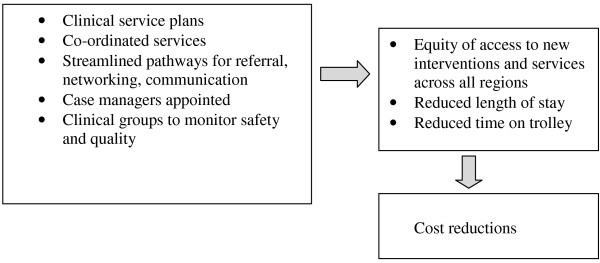
**Nominated service delivery and patient outcomes**.

A number of specific and concrete outcomes related to service delivery and patient outcomes were nominated by all stakeholder groups (Figure 1). These largely reflected a focus on improving patient journeys; standardising care through the provision of services that extended beyond the metropolitan area; reducing costs and monitoring quality.

Apart from a participant from the network driver group nominating patient satisfaction with new services arising from network innovations, for the most part improving care outcomes were defined in relation to service delivery objectives and in terms of impact measures such as length of stay, rather than in terms of specific clinical outcomes:

"They [the network] were able to prove statistically that they were actually reducing length of stay in the inpatient services, they were getting quicker turn around on their outpatient services, they were reducing the costs associated with the care of the patients in the community."

Only one network participant gave an account of patient outcomes in terms of achieving risk reductions or fulfilling a pre-determined target in relation to a specific clinical focus:

"I can say we've reduced the rate of aspiration pneumonia from 11 per cent in that quarter to zero in this quarter. That was huge."

Initiatives that addressed workforce development and clinical education were cited as important outcomes as they were thought to lead to other desirable outcomes such as job satisfaction, the development of clinical career paths and the retention of a stable clinical workforce that could carry forward network initiatives. Obtaining funding for clinical positions; scholarships and speciality post-graduate studies; clinical fellowship posts; and conference support schemes and the establishment of new postgraduate courses in clinical specialties were all cited as desirable network outcomes because they contributed to retention of the workforce and enabled professional pathways into obtaining specialist qualifications:

"We have been able to fund so many nurses to do post-graduate studies in neurology. So it's made stroke more of a career. In my opinion -- it's been a major retention for neurosurgery."

#### Implementing evidence based practice

The implementation of projects focused on evidence translation were considered important outcomes by all stakeholder groups because of their potentially significant value in improving patient outcomes. The implementation of clinical guidelines and protocols and audits of practice against evidence based benchmarks to demonstrate improvements were commonly nominated by the network groups as outcomes, as was the development of or adaptation of guidelines. However, one participant from the Senior policy-maker group mentioned that he was aware of networks that did not wholly embrace an evidence-based approach or based their initiatives on 'sources of evidence that I wouldn't find credible'.

A major outcome nominated by Senior health service managers and policy-makers was addressing clinical variation, with one Senior health service manager stating that: 'Success is if you get to the point of reducing clinical variation.' While networks were seen as being a useful vehicle to improve consistency and standards of care, involvement of networks in addressing clinical variation was seen as an outcome that had yet to be realised:

"Some people want to tackle it but whenever you take this up with clinicians, they all say, oh yes it's very important but it's not about me. Or, I'm different, or my patients are different, or my patients are sicker, blah, blah, blah."

Another desirable outcome, cited across all stakeholder groups, was the development of multidisciplinary research collaborations and research agendas to generate evidence and also to enable comparisons of patterns of practice in the different areas covered by a network, help to identify variations in practice and to assess the impact of network initiatives.

## Discussion

Through thematic analysis, this study has identified stakeholder views on the important conditions for establishing well-functioning and successful clinical networks within the broader health system and also captured what stakeholders think are desirable network outcomes. Our study provides insight into the views of those with strategic and health policy and planning responsibilities and those who work within or are involved in clinical networks. Although mainly broadly supportive of network initiatives, the views of those in the Senior policy-maker and health service manager groups were not as uniformly positive as those in the network groups. Concerns were mainly focused on the need for network workplans to be aligned with state health and local health services priorities and for there to be a formalisation of alliance between the networks and local health service managers in order for networks to improve clinical outcomes. Those in the network groups were mainly concerned about bureaucratic obstacles to progressing their initiatives and also with practical considerations around availability of resources and not being able to implement innovations.

However, there was a broadly shared vision across all stakeholder groups that effective structure, organisation and governance be in place for networks to be well-established and effective in achieving health system outcomes. Lack of formal governance, structure and organisational focus was widely thought to result in poorly organised and functioning networks with limited ability to realise their objectives and liaise strategically with within the broader strategic health arena. The necessary conditions to achieve this is represented by the sub-themes of *building relationships*; *leadership*; *strategic evidence-based workplans*; *adequate resources*; and *ability to implement and evaluate network initiatives *would help.

An important condition was the development of an inclusive and collegial network ethos with a strong focus on working for healthcare consumers. The importance for network development of involving a critical mass of clinicians in health care service planning and delivery and forming interdisciplinary and interorganisational collaborations for achieving health outcomes and practice change has been identified as important in other contexts [8,10,12,18]. Other critical ingredients for network success was strong multi-level leadership to engage clinicians, drive change and to implement clearly articulated evidence-based workplans. Other studies have highlighted the importance of leadership in order to effect positive change in health systems [1,12,14].

There was concordance amongst stakeholders that links with state health agencies and local health services were essential for establishing successful networks. However, tensions in relationships between some networks and health regions and health agencies was felt to constrain some network activities, although different reasons for this were nominated by the different stakeholder groups. For participants from network groups, there was concern that area health service boundary issues were a substantial barrier to realising the benefits of network innovations across a broad geographical area. While for those in the Senior health service manager group, the stated issue was that health managers were not formally involved in networks. Participants from all groups felt that these relationships would develop positively over time as networks became more established and more embedded within the health system. The feedback about the desirability of aligning network workplans with state health priorities on the one hand and objectives that may engage clinicians on the other hand, suggest that a balance needs to be struck to ensure projects are of interest to clinicians but also that resources are directed to innovations that will have the most impact on patient outcomes. In the early stages of network development, some quick gains from simple projects may be motivating and serve to keep clinicians on board for more complex projects.

Our findings converge with those in previous studies suggesting that there are a core set of preconditons that may be required by networks in different settings to ensure that they can deliver successful projects. Touati et al (2006) found a shared philosophy and vision to be important factors for success[12] and Nies et al. (2003) found that the main obstacles to network success were: competing interests and priorities; overlapping catchment areas and complexity due to the involvement of multiple levels of government [19]. A cross-sectional multiple case study of six managed clinical networks found that the three major determinants of successful networks were: professional dedication of network staff; legitimacy of the network and confidence of the staff and organisations involved [7].

The present study also reports what participants identified as desirable outcomes of successful networks. The two main themes, connecting and engaging and changing the landscape of care, reflected two broad categories of both process and 'hard' outcomes. The nominated outcomes of interprofessional collaboration and strategic external partnerships were frequently cited by those in the network groups as being as highly valued as achieving clinical outcomes. Interestingly, this reflected the perspective of both those who were associated with networks that had been relatively newly established, and those whose network had been operating for a number of years. The value of these 'relationship' outcomes is that they provide a foundation for achieving health service delivery and patient outcomes. That this type of outcome was regarded as highly desirable and was also nominated as a condition ('building relationships') indicates the strength of its importance to the formation and development of well-organised networks. However, this does not necessarily indicate the relative importance of this condition/outcome compared to others, as participants were asked to nominate those conditions and desirable outcomes that they felt were important, not to rank them.

The theme 'changing the landscape of care' reflected strong views about the value of networks in improving health services, patient outcomes and also the role of networks in developing and retaining an engaged clinical workforce through fellowships; post-graduate education, scholarships and conferences. However, some Senior health service managers and policy-makers expressed concerns about whether networks are well-placed to achieve some of the more ambitious outcomes, such as addressing clinical variation. However, the reasons for gaps between evidence and practice are complex requiring complex solutions [20] so the type of project required to achieve outcomes related to addressing clinical variation may be overly ambitious for networks to address in the early stages of the development of a network and/or without support from experienced researchers and other experts.

Related to this, it is noteworthy that in the course of the interviews, some participants, mainly from the network groups, expressed doubts about potential difficulties in applying a standardised formula of measuring success and outcomes across all networks. This suggests that while it may be possible to develop a core set of outcomes to which networks could aspire to, this suggests that network-specific outcomes which reflect networks different clinical foci and priorities, differing time periods over which networks have been established and available staffing and other resources are also needed. This is supported by literature that recommends that comparative studies of health service change strategies, such as clinical networks, should use a range of measures of success and impact to enable a more complete assessment of effectiveness [21,22].

Accessing a range of views on a relatively new initiative is useful for those with responsibilities for establishing and working with networks as it can identify both issues and possible solutions. The findings from this study point to the importance of ensuring that favourable conditions are in place to maximize the effectiveness of networks in achieving outcomes. Participants views indicated that being able to succeed in achieving network objectives and therefore achieving both process and hard outcomes is partly conditional on the right factors being in place in order to establish a solid foundation for networks to design evidence-based projects. Future research is warranted on whether networks with clearly defined and formal structure, organisation and governance compared to more informal network arrangements are more likely to achieve their outcomes.

### Strengths and limitations

The strengths of our study include the maximum variation sampling strategy that ensured that multiple perspectives were captured through in-depth interviews of informants from four groups related to clinical networks. The sample included both those directly involved in networks and those who were knowledegable about, but not directly involved in the day to day work of networks. Stakeholders were asked to give their general views; however many chose to contextualise their responses with reference to networks with which they had direct experience. The semi-structured interview technique allowed issues to be explored in a flexible manner. In keeping with the research method, interviewees were free to raise any issue that they felt were germane to the topic under investigation. As a result, it is believed that the information gathered was reflective of genuine concerns and views. The main limitation is that the sampled participants (network participants and drivers) came from 10 out of 20 networks. However, within these 10 there was good representation in terms of range of years that they had been operating, that is from two years upwards and also in terms of the variability of clinical area represented. In addition, it may be that the interviewees expressed publicly acceptable viewpoints. However, the interviews were anonymised and confidential in line with ethics requirements. In terms of transferability of results, clinical networks in NSW, indeed each network, may have their own unique culture and political environment that influence the responses of participants' directly involved in networks. These findings may therefore be most relevant for networks that have a similar model to that described here.

## Conclusions

This study has provided new knowledge on what key stakeholders believe are important conditions for successful networks and valuable outcomes of networks. The findings suggest that a systematic evaluation of barriers and facilitators prior to the establishment of networks should be undertaken to ensure that favourable conditions are in place to maximize the effectiveness of networks. The findings also suggest that tools assessing outcomes of clinical networks in future evaluative studies should be multi-dimensional, covering health care outcomes and process outcomes such as building relationships and interprofessional collaboration.

Importantly, stakeholders held the view that effective clinical networks could realise significant benefits for healthcare systems and patient care as long as the right conditions were in place. This makes networks well-placed to work collaboratively alongside health authorities to deliver population health goals. These findings are likely to be generally applicable to other similarly organised networks and studies of those networks.

## Competing interests

The authors declare that they have no competing interests.

## Authors' contributions

All authors contributed to the study's conceptualisation. EM, SM, GG and MH finalised the study design. EM, SM, GG and MH prepared the framework of this manuscript. EM analysed the data and led revisions of the paper. MHaertsch, CP and PC advised on protocol details including recruitment and provided input into the content of the manuscript. All authors read and approved the final manuscript.

## Pre-publication history

The pre-publication history for this paper can be accessed here:

http://www.biomedcentral.com/1472-6963/12/49/prepub
